# Assessing the Feasibility of Studying Awareness of a Digital Health Campaign on Facebook: Pilot Study Comparing Young Adult Subsamples

**DOI:** 10.2196/37856

**Published:** 2022-08-29

**Authors:** Shreya Tulsiani, Megumi Ichimiya, Raquel Gerard, Sarah Mills, Jeffrey B Bingenheimer, Elizabeth C Hair, Donna Vallone, W Douglas Evans

**Affiliations:** 1 Schroeder Institute Truth Initiative Washington, DC United States; 2 Department of Prevention and Community Health Milken Institute School of Public Health The George Washington University Washington, DC United States; 3 Global Institute of Public Health New York University New York City, NY United States; 4 Department of Health, Behavior and Society Johns Hopkins Bloomberg School of Public Health Baltimore, MD United States

**Keywords:** campaign evaluation, outcome evaluation, young adults, social marketing, health communications, tobacco control and policy, health campaign, youth, Facebook, digital media

## Abstract

**Background:**

Mass media campaigns for preventive health messaging have been shown to be effective through years of research. However, few studies have assessed the effectiveness of campaigns on digital media, which is currently how youths and young adults are primarily consuming media. In particular, a platform that can accurately assess exposure to digital messaging in a real-life setting has yet to be developed.

**Objective:**

This study examines the feasibility of a unique survey platform, Virtual Lab, to conduct a study on exposure to a media campaign within Facebook using a chatbot-style survey administration technique.

**Methods:**

Virtual Lab is a survey platform that was used to recruit and survey participants within Facebook and Facebook Messenger, respectively. We created a Facebook business account with 2 Facebook pages: one for recruitment and disseminating the survey and the other one for serving the target advertisements. Pre- and postexposure surveys were administered via Facebook Messenger using a chatbot-style questionnaire 1 week apart. During this time, the target advertisements were shown to participants who completed the pre-exposure survey. The total time from recruitment to completion of the postexposure survey was 13 days, and incentive costs were US $10 per participant. Survey data were compared between those who completed both pre- and postexposure surveys and those who only completed the pre-exposure survey; that is, those who were lost to follow-up. The demographics of the complete cases were also compared to the US census data.

**Results:**

A total of 375 Facebook users aged between 18 and 24 years met eligibility requirements and consented to the study, which consisted of complete cases (n=234) and participants lost to follow-up (n=141). A few differences between complete cases and participants lost to follow-up were observed. Regarding gender, complete cases comprised 40.2% males and 59.4% females, and among participants lost to follow-up, 44.0% were male and 50.4% were female (*P*=.003). Differences were also observed for e-cigarette use status, where a greater number of current users and fewer past and never users were lost to follow-up than complete cases (*P*=.01).

**Conclusions:**

The use of Virtual Lab yielded a diverse sample quickly and cost-effectively. Demographic characteristics of participants who completed the study and those who were lost to follow-up were similar, indicating that no biases were caused by the platform during recruitment or testing. This study suggests the feasibility of the Virtual Lab survey platform for studies of media campaign exposure within Facebook. This platform can advance health campaign research by providing more accurate data to inform digital messaging.

## Introduction

Mass media campaigns have been shown to be effective in preventive public health efforts, such as tobacco countermarketing [1,2]. Media campaigns have adapted to using digital media to disseminate health messages, particularly among target audiences that include youth and young adults. Having sufficient exposure of media campaigns is important to measuring their effectiveness [3]. However, a feasible platform to accurately assess exposure, awareness, and outcomes of digital messaging in a real-life setting is yet to be developed, which would ultimately help health communication researchers execute more accurate methodology for campaign evaluation and inform audience segmentation.

In recent years, the transition from a traditional media landscape (TV, newspaper, etc) to consuming digital media via social platforms and streaming services has been largely driven by youths and young adults [4]. With 81% of teens now using social media and more than 33% using social media sites multiple times in an hour, digital media has undoubtedly become a large part of youths’ and young adults’ lives [5,6]. A majority of 18-29-year-olds use social media platforms such as TikTok, Instagram, and YouTube, and 70% of 18-29-year-olds use Facebook [7]. This transition has occurred at a faster pace than research on how to measure campaign exposure in a digital landscape.

While TV uses standardized gross rating points, the fragmentation of digital media and the way in which people experience digital media have made it complex to determine a standardized encompassing metric. In digital media, advertising is often skippable and perceived as more of a disturbance [8,9], and the obstacles of data privacy make it complicated to obtain or create metrics that are standardized across platforms and parsimonious. There is little research on exposure of media campaigns in a real-life digital setting, likely owing to limited options of viable platforms [10,11]. An increasing number of studies are being conducted within social media, specifically Facebook, to recruit participants [12-14], as well as to assess the feasibility of using Facebook to reach and survey youths and young adults about the use of tobacco and other substances [15,16].

Our pilot study utilized Virtual Lab, a platform that enables research studies to be conducted on Facebook. Recruitment of participants and exposure to target advertisements occur on Facebook. The surveys are disseminated within Facebook Messenger using a chatbot questionnaire, which has been shown to increase user engagement, user satisfaction, and data quality over traditional web surveys [17-19]. The feasibility of Virtual Lab would allow for quick recruitment of participants and delivery of results, as well as easy navigation of a dashboard during data collection, while remaining cost-effective. The ability to conduct research within the same platform on which campaigns are actively being conducted is important to the progress of campaign evaluation research. The objective of this study was to determine the feasibility of the Virtual Lab platform to recruit participants and assess awareness of an anti–e-cigarette health campaign on Facebook.

## Methods

### Recruitment

**Figure 1:**
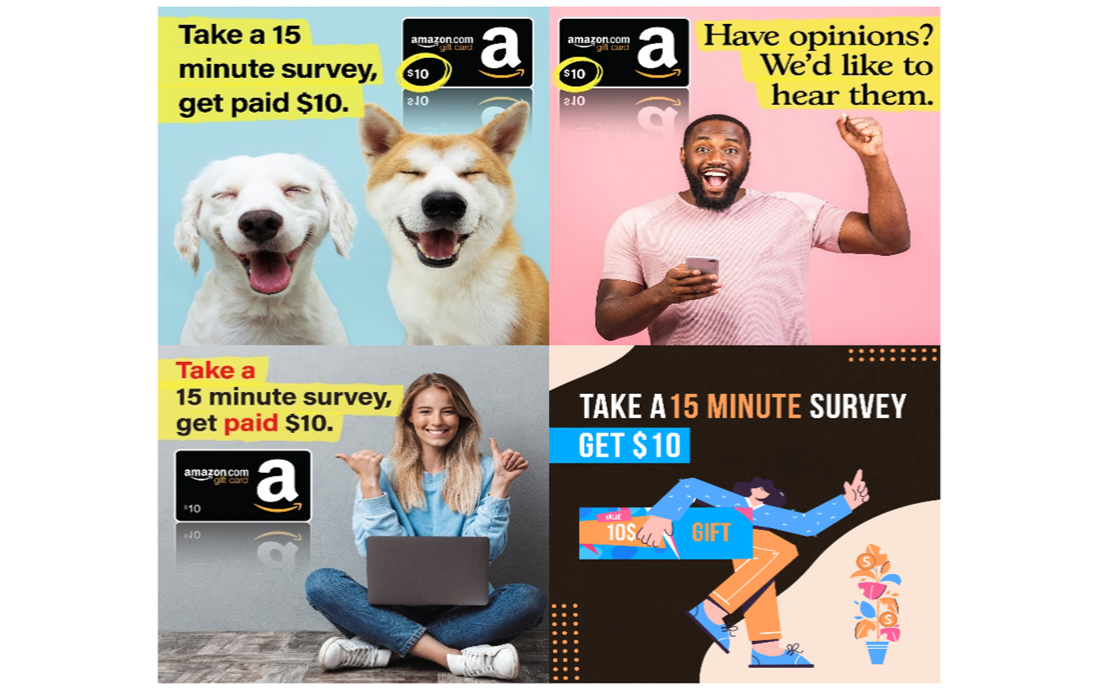
Recruitment advertisements shown on potential participants’ Facebook pages.

Virtual Lab is a survey platform used to recruit and survey participants within a social media platform, and is owned and run independently of social media companies [20]. To utilize what Virtual Lab has to offer, specifically within Facebook, the first step was to create a Facebook business account. To prevent potential biases, the account was given a general name, “Digital Health Research.” Under this account, we created a Facebook page, “Digital Media Experiment,” to host advertisements for recruitment purposes. In order to demonstrate the credibility of the page, we posted relevant content and acquired likes. We used this Facebook Page to run recruitment advertisements during August 5-12, 2021. The recruitment advertisements were shown to our target population of people aged 18-24 years and located in the United States. The two recruitment advertisements used in this feasibility study (Figure 1) were designed using the 99designs website. The advertisements used the text “Take a 15 minute survey, get paid $10.” After participants clicked on the study’s advertisement, they were sent a message via Facebook Messenger inviting them to participate in the study.

Under the same business account, we created another Facebook page named “Consumer Consciousness,” which was solely used to run the target advertisements on the enrolled participants’ Facebook Newsfeeds during August 20-26, 2021. We created this second Facebook Page with a different name to prevent biases.

### Ethical Considerations

Privacy measures include encryption of data that are stored and in transmission. Before beginning the pre-exposure survey, participants were sent messages regarding the topic of the survey, compensation, privacy measures, and contact information if they had any questions. Following those messages, the participants were sent a message asking for consent for their participation in the study and if they would like to continue. All studies were reviewed and approved by the institutional review board at George Washington University (NCR202837).

### Survey Implementation

The pre- and postexposure surveys were completed using a survey platform called Typeform, which is supported by Virtual Lab. After designing the surveys in Typeform, they were linked to our Facebook business account and pages using the Virtual Lab interface. Typeform used our “Digital Media Experiment” page to send automated messages through Facebook Messenger, similar to a chatbot, to participants who clicked on the recruitment advertisements. Through Typeform, we were able to create logic jumps based off participants’ responses and confirm that they met the recruitment criteria. If participants did not meet the eligibility criteria, they were thanked for their participation and the survey ended. If the participants met the eligibility criteria, they were allowed to continue the survey.

After participating in the pre-exposure survey administered by the “Digital Media Experiment” page, respondents randomized to be exposed were shown the target advertisement via the “Consumer Consciousness” page in their Facebook Newsfeeds. All participants were invited to take the postexposure survey a week after completion of the pre-exposure survey. The postexposure survey was also administered by the “Digital Media Experiment” page via Facebook Messenger.

### Measures

The pre-exposure survey consisted of questions to determine demographic characteristics and e-cigarette status. Questions determining demographics included age, gender, sexual orientation, combined race and ethnicity, and perceived financial status. e-Cigarette use status was determined through 2 questions: an ever-use question, “Have you ever tried using any e-cigarette/vape (even 1 or 2 puffs)?” with response of “yes” or “no.” For those who responded with “yes,” there was a current-use question, “During the past 30 days, on how many days did you use an e-cigarette (even 1 or 2 puffs)?” where they could respond with 0-30 days. Respondents were classified as never users if they responded with “no” to the ever-use question, as past users if they responded with “yes” to the ever-use question and 0 to the current-use question, and as current users if they responded with “yes” to the ever-use question and >1 days to the current-use question.

Intentions to use e-cigarettes was determined by asking the question, “Do you think you will use an e-cigarette (even 1 or 2 puffs) in the next year?” Answer choices were “definitely not,” “probably not,” “probably yes,” and “definitely yes,” where “definitely not” was coded as no intentions to use and all other choices were coded as having intentions to use. Intentions to quit using e-cigarettes was only asked of current users: “Are you seriously thinking about quitting e-cigarettes/vapes for good?” The responses were dichotomized where “No, I am not thinking about quitting” was coded as no quitting intentions and the following were coded as having intentions to quit: “Yes, but not within the year,” “Yes, within the year,” “Yes, within the next 6 months,” “Yes, within the next 30 days,” and “I’ve already quit.”

The post-survey included questions for e-cigarette use status and intentions to use and quit, as listed above, as well as questions about the target advertisement they were exposed to between taking the pre- and postexposure surveys. These questions were asked in the pilot study to ensure feasibility for the proceeding full study. Participants were first shown an advertisement they were exposed to and asked if they can see and hear the video. If they responded with “no,” they were directed to the end of the survey. Those who responded with “yes” were asked how many times they have seen the advertisement, receptivity questions on what they thought of the advertisement, and about actions they would take after seeing the advertisement. This series of questions on the target advertisement were then repeated for the second target advertisement.

### Statistical Analysis

A series of descriptive analyses were conducted to examine the feasibility of recruitment methods and the diversity of samples. Survey data were compared between complete cases and participants lost to follow-up and among different e-cigarette use status groups. Complete cases were defined as participants who completed the survey through the last question in the postexposure survey. Loss to follow-up was defined as participants having been lost at any point before the last question in the postexposure survey.

In analysis 1, we compared sample demographics and use status between complete cases and participants lost to follow-up. For continuous measures, means and SDs were obtained and *t* tests were conducted to compare differences between groups. For categorical measures, frequencies and proportions were obtained and Pearson chi-square tests were conducted. The analysis included the number of missing participants to observe the difference between complete cases and participants lost to follow-up.

In analysis 2, we compared sample demographics with the US national survey data. Race and ethnicity were compared by sex between the US population and complete cases collected in this study. The US national demographics for the population aged 18-24 years were retrieved from the US Census Bureau as of 2019 (the most recently available year). We conducted chi-square tests to evaluate the difference of proportions of these demographics between the US population and the study sample. Analyses were conducted using Stata SE 15 (StataCorp) and Excel (Microsoft Inc).

### Results 

#### Recruitment and Target Advertisements

**Figure 2:**
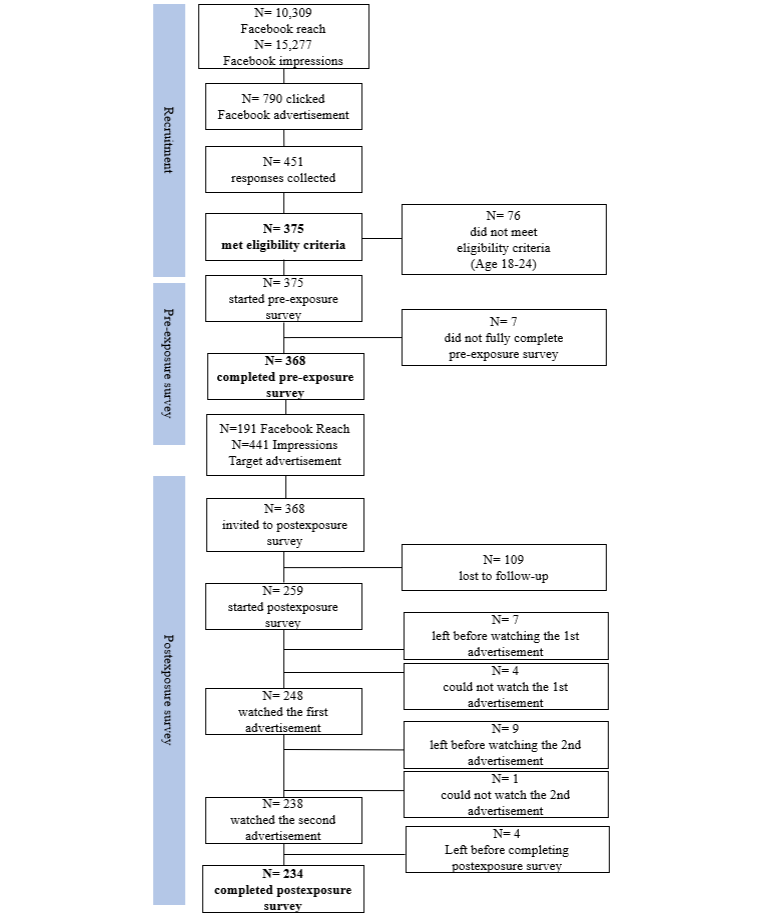
Flowchart of sample recruitment and retention.

The study’s Facebook recruitment advertisements had a reach of 10,309, which is defined as the number of unique individuals who saw the advertisement at least once. The recruitment advertisement generated 15,718 impressions—that is, the number of times the advertisement was displayed on a person’s screen—and was clicked a total of 790 times. The percentage of times a person saw the recruitment advertisement and clicked on it, or the Link-Click-Through Rate, was 4.77%. The study’s Facebook target advertisements had a reach of 191 unique individuals and generated 441 impressions. The target advertisements were played a total of 353 times and were played to 100% of their length only 11 times. The largest drop after any play time to 25% of the total advertisement length was from 25% to 50% of their length (70 and 29 times, respectively). The advertisements were played to 75% of their length 19 times. A visual representation of the in-platform recruitment data is shown in Figure 2.

The subsample of participants lost to follow-up consisted of participants who began the pre-exposure survey but did not finish (n=7), completed the pre-exposure survey but did not begin the postexposure survey (n=109), started the postexposure survey but did not watch the first or second advertisement (n=21), and watched both advertisements but did not complete the postexposure survey (n=4). If they did not watch either advertisement in the postexposure survey, it could have been because they left the survey or responded that they could not see or hear the advertisement.

#### Sample Characteristics

Sample demographics are summarized in Table 1 and organized by the total sample (n=375) and subsamples of those who completed both surveys (n=234) and those lost to follow up (n=141). Participants included 375 Facebook users aged 18 to 24 years. They were on average 21 (SD 2.0) years old, 56.0% female, and racially and ethnically diverse (45.6% non-Hispanic White, 12.0% Hispanic, 10.1% non-Hispanic Black or African American, or 23.7% non-Hispanic Asian). Of them, 45.6% were never users, 20.5% were past users, and 33.3% were current users.

**Table 1:** Demographics and e-cigarette use status between complete cases and participants lost to follow-up (N=375).

Variables			Total participants		Complete cases (n=234)		Participants lost to follow-up (n=141)		*P* value	
Age (years), mean (SD)			21.0 (2.0)		21.1 (2.0)		20.8 (2.0)		.14	
**Gender, n (%)**										.003
	Male	156 (41.6)		94 (40.2)		62 (44.0)				
	Female	210 (56.0)		139 (59.4)		71 (50.4)				
	Other or missing	9 (2.4)		1 (0.4)		8 (5.7)				
**Sexual orientation, n (%)**										.69
	Heterosexual	240 (64.0)		155 (66.2)		85 (60.3)				
	Homosexual	29 (7.7)		17 (7.3)		12 (8.5)				
	Bisexual	63 (16.8)		36 (15.4)		27 (19.1)				
	Other or missing	43 (11.5)		26 (11.1)		17 (12.1)				
**Race and ethnicity, n (%)**										.28
	Non-Hispanic White	171 (45.6)		102 (43.6)		69 (48.9)				
	Hispanic	45 (12.0)		30 (12.8)		15 (10.6)				
	Non-Hispanic Black or African American	38 (10.1)		22 (9.4)		16 (11.3)				
	Non-Hispanic Asian	89 (23.7)		63 (26.9)		26 (18.4)				
	Other/missing	32 (8.5)		17 (7.3)		15 (10.6)				
**Financial status, n (%)**										.11
	Live comfortably	119 (31.7)		75 (32.1)		44 (31.2)				
	Meet needs with a little left over	135 (36.0)		94 (40.2)		41 (29.1)				
	Just meet basic expenses	69 (18.4)		39 (16.7)		30 (21.3)				
	Do not meet basic needs	21 (5.6)		11 (4.7)		10 (7.1)				
	Decline to answer/missing	31 (8.3)		15 (6.4)		16 (11.3)				
**e-Cigarette use status, n (%)**										.01
	Never user	171 (45.6)		112 (47.9)		59 (41.8)				
	Past user	77 (20.5)		56 (23.9)		21 (14.9)				
	Current user	125 (33.3)		66 (28.2)		59 (41.8)				
	Missing	2 (0.5)		0 (0.0)		2 (1.4)				

#### Analysis 1

The proportions for gender and use status differed between complete cases and participants lost to follow-up. Complete cases comprised 40.2% males and 59.4% females, whereas participants lost to follow-up comprised 44.0% males and 50.4% females (*P*=.003). Based on chi-square analysis, the statistical difference found for gender was driven by the “other/missing” category. Complete cases comprised 47.9% never users, 23.9% past users, and 28.2% current users, whereas participants lost to follow-up comprised 41.8% never users, 14.9% past users, and 41.8% current users (*P*=.01); the largest difference was observed among current users. Age, sexual orientation, race and ethnicity, and financial status did not significantly differ between groups.

#### Analysis 2

The proportions of gender and race and ethnicity in complete cases in the study sample were significantly different from the US census data, as shown in Table 2. The sample comprised 40.3% males and 59.7% females, whereas the US census data set comprised 51.3% males and 48.7% females (χ^2^_1,233_=11.2, *P*<.001). The sample included 43.4% Non-Hispanic White, 12.9% Hispanic, 9.4% Non-Hispanic Black or African American, and 27.0% Non-Hispanic Asian participants. This was significantly different from the US census data set that comprised 53.7% Non-Hispanic White, 22.1% Hispanic, 14.3% Non-Hispanic Black or African American, and 5.5% Non-Hispanic Asian participants (χ^2^_4,233_=218.4, *P*<.001).

**Table 2:** Demographics between US national survey and complete cases among 18-24–year-olds.

Race and ethnicity	US national survey^a^			Complete cases		
	Total sample, %	Males, %	Females, %	Total sample, %	Males, %	Females, %
Non-Hispanic White	53.7	51.4	48.6	43.4	43.6	56.4
Hispanic^b^	22.1	51.5	48.5	12.9	43.3	56.7
Non-Hispanic Black or African American	14.3	50.7	49.3	9.4	9.1	90.9
Non-Hispanic Asian	5.5	50.8	49.2	27.0	49.2	50.8
Other/missing	4.4	50.5	49.5	7.3	23.5	76.5
Total	100	51.3	48.7	100	40.3	59.7

^a^Source: American Community Survey 5-Year Estimates–Public Use Microdata Sample 2019.

^b^Derived as the difference between total and non-Hispanic counts from the US Census Bureau.

## Discussion

### Principal Findings

To our knowledge, this is the first study to examine the utility of Virtual Lab within the real-life setting of Facebook as a viable platform for media campaign awareness studies. The results of this pilot study provide support for continued use of the Virtual Lab platform to recruit participants and obtain data from a nationwide sample.

We found that recruitment yielded a diverse sample, consistent with other convenience samples [10,21], and it was carried out cost-effectively and quickly. Within 1 week of recruitment and 1 week of conducting the study, 375 respondents participated in the study with a total of 234 to complete the study. Including the US $5 Amazon e-gift cards for each survey, the total cost for incentives per respondent was approximately US $10 [22]. There were additional costs for executing the study and recruitment, which vary on the basis of project goals and sample size; expenses remained lower than those on other survey platforms. Overall, this is a low-cost data collection option with great potential for high reach and customized sampling by respondent characteristics via longitudinal panel research on a social media platform.

Most demographic characteristics were similar between those who completed the study and those who were lost to follow-up, indicating that the platform is not causing biases in recruitment or testing to result in certain groups dropping out of the study at greater rates than others. Chi-square analysis showed a difference in gender between complete cases and participants lost to follow-up. This was also observed when comparing the samples to the US census data. However, a resulting sample with a greater proportion of females than males is commonly seen in surveys using a convenience sample [10,21]. Although the sample lost to follow-up shows a higher rate of current use status than that of the complete cases sample, the latter consists of a significant amount of past and current e-cigarette users, comprising over 50% of the sample. e-Cigarette use status in the completion sample is also similar to nationwide prevalence numbers, deeming the sample to still be valuable and representative.

### Limitations

Although the results support the feasibility of the Virtual Lab platform, there are some limitations to this study. First, a large proportion (37.6%) of the eligible starting sample of the pilot study was lost to follow-up. Efforts to bolster retention are being made for the proceeding study, including increasing compensation for participation and speed of distribution of compensation. Second, this study only examined the feasibility of Virtual Lab on Facebook. Thus, the feasibility of Virtual Lab on other social media platforms, such as Instagram, will require future research once it becomes available. Third, there were limitations in the ways to ask questions within Facebook Messenger using the Typeform platform. One of these limitations includes the inability to select more than one response for a question, such as identification of race and ethnicity. In order to address this limitation, we programmed the survey to repeat questions where multiple-choice responses would normally be available until the participant indicated that they had selected all relevant responses. Another limitation we encountered when converting the Typeform survey to Facebook Messenger was the inability to boldface words. 

Digital health is a growing field, but there has been relatively little research using social media platforms to recruit participants, deliver interventions, and collect data. The ability to follow up with participants over time and collect data in a low-cost, rapid, and relatively low-burden manner offers tremendous potential for social media health research and interventions. It can lead to more effective campaign interventions that aim to improve youth and young adults’ health behaviors, such as substance use prevention. This study suggests that such intervention studies are feasible and may be a valuable tool for researchers. Future studies should include randomized controlled trials in real-world settings on Facebook and other social media, provide social media stimuli aimed at health behavior change, and follow participants over time to evaluate outcomes.

### Conclusions

The development and use of a platform that allows for experimentation within social media platforms is essential for the progress of mass media campaign evaluation research. Virtual Lab, a new cost-effective platform that allows for customized recruitment and longitudinal follow-up of participants and execution of survey research on Facebook, has shown to be feasible for media campaign awareness studies. Importantly, with the use of Virtual Lab, research can result in more accurate data to inform health campaigns and their dissemination.
